# Quantitative 3D assessment of marginal impaction volume in posterior wall acetabular fractures: a pilot study

**DOI:** 10.1186/s13018-026-06669-8

**Published:** 2026-01-17

**Authors:** Fatih Emre Topsakal, Ekrem Özdemir, Nasuhi Altay, Fahri Berkay Ağ, Yavuz Şahbat, Esra Demirel

**Affiliations:** Department of Orthopedics and Traumatology, Erzurum City Hospital, 25240 Erzurum, Turkey

**Keywords:** Acetabular fractures, Marginal impaction, Volumetric analysis, 3D imaging, Prognosis

## Abstract

**Background/Objective:**

Marginal impaction in acetabular posterior wall fractures significantly influences long-term prognosis, yet current assessment methods remain qualitative. This study introduces quantitative volumetric analysis using three-dimensional computed tomography to evaluate the prognostic significance of impaction volume—representing the first volumetric quantification of marginal impaction in the literature.

**Methods:**

Twenty-two patients with acetabular posterior wall fractures and marginal impaction treated between May 2021 and October 2023 at a tertiary trauma center were retrospectively analyzed. Preoperative computed tomography scans were processed using 3D Slicer-5.8.1 software to measure impacted fragment volumes and impaction volume-to-acetabular volume ratios. Functional outcomes were assessed using Harris Hip Score (HHS) and Modified Merle d'Aubigné-Postel Score (MMAS) at 6 months and 2 years. Statistical analysis included correlation analysis, receiver operating characteristic curves, and multivariate regression to identify prognostic factors.

**Results:**

Mean patient age was 45.3 ± 16.8 years (range 23–74) with mean body mass index of 26.4 ± 3.8 kg/m^2^. Mean impaction volume was 1,847.3 ± 1,124.6 mm^3^ (range 89–3,842 mm^3^). Receiver operating characteristic curve analysis identified an exploratory threshold of 2000 mm^3^ for predicting poor functional outcomes, with area under the curve 0.91 (95% CI: 0.78–1.00, p < 0.001). Patients with impaction volumes > 2000 mm^3^ (n = 8, 36.4%) demonstrated significantly worse functional outcomes at 2 years compared to those with ≤ 2000 mm^3^ (HHS: 68.3 ± 10.8 vs 88.5 ± 7.2, p < 0.001; MMAS: 13.4 ± 2.3 vs 17.6 ± 1.2, p < 0.001). Post-traumatic osteoarthritis developed in 9 patients (40.9%), with significantly higher rates in the high-volume group (75.0% vs 21.4%, p = 0.012). Impaction volume showed strong negative correlation with functional scores at 2 years (HHS: r = -0.782, p < 0.001; MMAS: r = -0.758, p < 0.001). The impaction volume-to-acetabular volume ratio averaged 5.12 ± 3.15% and demonstrated similar prognostic value.

**Conclusions:**

This study presents the first quantitative volumetric measurement of marginal impaction in acetabular fractures, which may offer improved prognostic discrimination compared to qualitative assessment in this cohort. An exploratory threshold of 2000 mm^3^, derived from receiver operating characteristic analysis, appears to stratify patients into different risk categories in this cohort for poor functional outcomes. This objective measurement tool may enhance surgical decision-making and patient counseling in acetabular fracture management. Future multicenter studies are needed to validate this threshold and establish standardized volumetric protocols.

## Introduction

Acetabular fractures represent complex orthopedic injuries that pose significant challenges in achieving optimal long-term outcomes [1, 2]. Among the various fracture patterns, posterior wall fractures with associated marginal impaction are particularly problematic, occurring in 30–42% of surgically treated cases and carrying substantial risk for post-traumatic complications [3, 4]. Marginal impaction, first described by Letournel and Judet, involves crushing of the articular surface and underlying subchondral bone into the softer cancellous bone of the acetabulum, resulting in joint incongruity and instability [5].

The clinical significance of marginal impaction lies in its established association with poor prognosis when inadequately addressed during surgical intervention [6, 7]. Current assessment methods rely primarily on qualitative visual interpretation of computed tomography (CT) images, with descriptive terms such as "small" or "large" impaction lacking objectivity and reproducibility [8]. While three-dimensional CT reconstruction has enhanced fracture visualization and classification accuracy, the quantitative measurement of impacted bone volume remains unexplored in clinical practice [9, 10].

The quality of surgical reduction, as assessed by Matta's radiological grading system, remains the most critical prognostic factor for acetabular fractures [11]. However, this two-dimensional measurement approach fails to capture the three-dimensional nature of the articular defect or the volume of impacted bone requiring elevation and grafting [12, 13]. The volumetric extent of impaction may offer additional prognostic information beyond traditional linear measurements, particularly given the complex three-dimensional geometry of these injuries.

Recent advances in medical image computing, particularly open-source platforms such as 3D Slicer, have enabled precise quantitative volumetric analysis of anatomical structures from CT data [14]. These tools offer the potential to transform subjective assessment of marginal impaction into objective, reproducible measurements that could enhance prognostic accuracy and surgical decision-making. Notably, a comprehensive literature review reveals that no previous studies have quantified marginal impaction volume or established volumetric thresholds for prognostic stratification—the existing literature relies exclusively on linear measurements (step-off > 2 mm) and qualitative descriptors [15].

The primary objective of this study was to evaluate the prognostic significance of quantitatively measured marginal impaction volume in patients with acetabular posterior wall fractures, representing, to our knowledge, an exploratory attempt at volumetric quantification of this injury feature. We hypothesized that larger impaction volumes would correlate with poorer functional outcomes and higher rates of post-traumatic complications. Secondary objectives included establishing a clinically relevant threshold value for impaction volume using receiver operating characteristic (ROC) analysis, introducing a novel impaction volume-to-acetabular volume ratio as a normalized metric, and evaluating the correlation between volumetric measurements and established outcome measures at multiple time points.

## Materials and methods

### Study design and patient selection

This retrospective observational study was conducted at a single tertiary trauma center following approval by the Institutional Review Board (Erzurum Medical Faculty Scientific Research Ethics Committee for Clinical Research; approval number: 2025/10–261) and in accordance with the principles of the Declaration of Helsinki. All patients who underwent surgical treatment for posterior wall acetabular fractures with associated marginal impaction between May 2021 and October 2023 were identified through the institutional trauma registry and electronic medical record system. Because this was a retrospective observational study based on routinely collected clinical data, prospective clinical trial registration was not required according to institutional and national regulations. *Informed consent to participate was obtained from all patients for the use of their radiographs and clinical data in this research, in accordance with institutional guidelines and the Declaration of Helsinki.*

Inclusion criteria: (1) Age ≥ 18 years; (2) Isolated acetabular posterior wall fracture with radiographically evident marginal impaction; (3) Surgical treatment within 21 days of injury; (4) Availability of high-resolution preoperative CT scans (slice thickness ≤ 1.5 mm); (5) Minimum 24-month clinical and radiographic follow-up.

Exclusion criteria: (1) Pathological fractures; (2) Previous ipsilateral hip surgery or fracture; (3) Associated acetabular fracture patterns (transverse, both-column, etc.); (4) Concurrent femoral head or neck fractures requiring surgical intervention; (5) Incomplete imaging or clinical follow-up data; (6) Active malignancy or metabolic bone disease; (7) Cognitive impairment preventing reliable outcome assessment.

### Imaging protocol and volumetric analysis

All patients underwent standardized CT imaging using a 64-slice multidetector CT scanner (Siemens SOMATOM Definition AS + , Erlangen, Germany) with the following parameters: 120 kVp, 200–400 mAs, 0.75–1.25 mm slice thickness, and 0.5 mm reconstruction interval. Axial images were obtained from the L3 vertebral body to the mid-femur with patients in supine position.

**Fig. 1:**
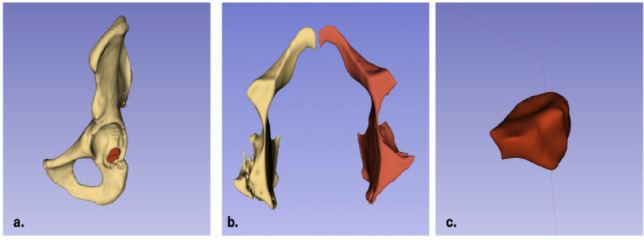
**a** Three-dimensional reconstruction image obtained using 3D Slicer software, demonstrating segmentation of the marginal impaction area (highlighted in red) on the posterior wall of the acetabulum. **b** Bilateral acetabular volumes were measured, and the collapsed segment volume was validated by calculating the volumetric difference between both sides. **c** The impacted segment volume was measured as 1620 mm^3^, confirmed through both manual segmentation and volumetric comparison between the intact and fractured acetabula

Volumetric analysis was performed using 3D Slicer version 5.8.1 (www.slicer.org), an open-source medical image computing platform [14, 16]. The workflow consisted of the following steps: (1) Data Import: DICOM files were loaded into 3D Slicer and converted to NRRD format for processing; (2) Image Preprocessing: Bone window settings (400/1000 HU) were applied for optimal visualization; (3) Impaction Segmentation: The impacted bone fragment was manually segmented using the "Segment Editor" module by an experienced orthopedic surgeon (> 10 years experience) on axial slices at 1 mm intervals. The boundaries were defined as the displaced osteochondral fragment depressed below the level of the intact articular surface, encompassing both the cortical and cancellous components of the impacted segment; (4) Acetabular Volume Measurement: The entire acetabular cavity was segmented from the sourcil (acetabular roof) to the inferior margin of the acetabulum to calculate total acetabular volume, enabling calculation of the impaction volume-to-acetabular volume ratio; (5) Quality Control:Accuracy of segmentation was verified using three-dimensional reconstruction and multiplanar review. The volumetric difference was measured relative to the intact acetabulum; (6) Volume Calculation: The "Segment Statistics" module automatically calculated total volumes in cubic millimeters (Fig. 1).

To ensure measurement reliability, 25% of cases were independently analyzed by a second observer (orthopedic trauma fellow), and intraobserver reliability was assessed by repeat measurement of 10 randomly selected cases after a 2-week interval.

### Surgical technique and postoperative management

**Fig. 2:**
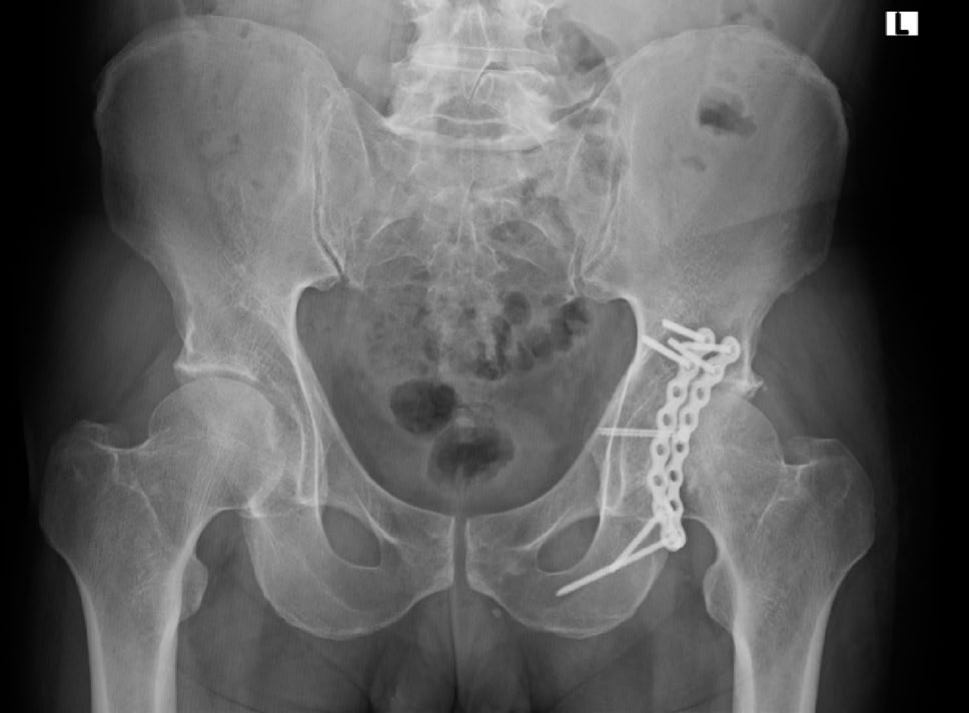
Postoperative imaging following Kocher-Langenbeck approach for posterior wall acetabular fracture with marginal impaction. Impacted osteochondral fragments were elevated, residual voids were filled with autograft or allograft bone, and fixation was achieved using reconstruction plates and lag screws under fluoroscopic control

All surgeries were performed by fellowship-trained orthopedic trauma surgeons using a standardized posterior (Kocher-Langenbeck) approach. The surgical protocol included: (1) Fracture Exposure: Complete visualization of the posterior wall and impacted fragments; (2) Impaction Reduction: Gentle elevation of impacted osteochondral fragments using curved osteotomes; (3) Bone Grafting: Autologous iliac crest bone graft or allograft chips to fill residual void space; (4) Internal Fixation: Reconstruction plates and/or lag screws as appropriate; (5) Stability Assessment: Intraoperative fluoroscopic evaluation of hip stability and reduction quality (Fig. 2).

Postoperative management followed institutional protocols with progressive weight-bearing as tolerated beginning at 8–12 weeks, depending on fracture stability and patient factors.

### Outcome assessment

Clinical and radiographic evaluations were performed at 6 weeks, 3 months, 6 months, 12 months, and 2 years postoperatively. Functional outcomes were assessed using two validated instruments at 6 months and 2 years: (1) Harris Hip Score (HHS): 100-point scale assessing pain, function, range of motion, and deformity [17, 18]; (2) Modified Merle d'Aubigné-Postel Score (MMAS): 18-point scale evaluating pain, mobility, and walking ability [19]. Outcomes were categorized as excellent (HHS > 90, MMAS 17–18), good (HHS 80–90, MMAS 15–16), fair (HHS 70–79, MMAS 13–14), or poor (HHS < 70, MMAS < 13).

Radiographic Assessment: (1) Reduction quality assessed using Matta's criteria on anteroposterior pelvis and Judet views [20]; (2) Post-traumatic osteoarthritis graded according to Brooker classification [21]; (3) Avascular necrosis evaluation using Ficat and Arlet staging [22].

Complications: (1) Infection (superficial or deep); (2) Nonunion or malunion; (3) Heterotopic ossification (Brooker classification); (4) Sciatic nerve injury; (5) Need for conversion to total hip arthroplasty.

### Statistical analysis

Statistical analyses were performed using IBM SPSS Statistics version 28.0 (IBM Corp., Armonk, NY). Data distribution was assessed with the Shapiro–Wilk test. Continuous variables are presented as mean ± standard deviation or median with interquartile range, as appropriate, while categorical variables are reported as frequencies and percentages.

Receiver operating characteristic (ROC) curve analysis was used to determine an exploratory threshold for marginal impaction volume in predicting poor functional outcomes, defined as a Harris Hip Score < 80 or a Modified Merle d’Aubigné–Postel Score < 15 at the 2-year follow-up. The optimal cut-off value was identified using the Youden index. Group comparisons were conducted using the independent samples t-test or Mann–Whitney U test, and correlations were analyzed using Pearson or Spearman coefficients depending on data distribution.

Multivariate linear regression analysis was performed to identify independent predictors of functional outcomes, and logistic regression analysis was applied for binary outcomes. Time to conversion to total hip arthroplasty was evaluated using Kaplan–Meier survival analysis. Interobserver and intraobserver reliability of volumetric measurements were assessed using intraclass correlation coefficients, with agreement further evaluated by Bland–Altman analysis. Statistical significance was set at p < 0.05.

## Results

### Patient demographics and injury characteristics

**Fig. 3:**
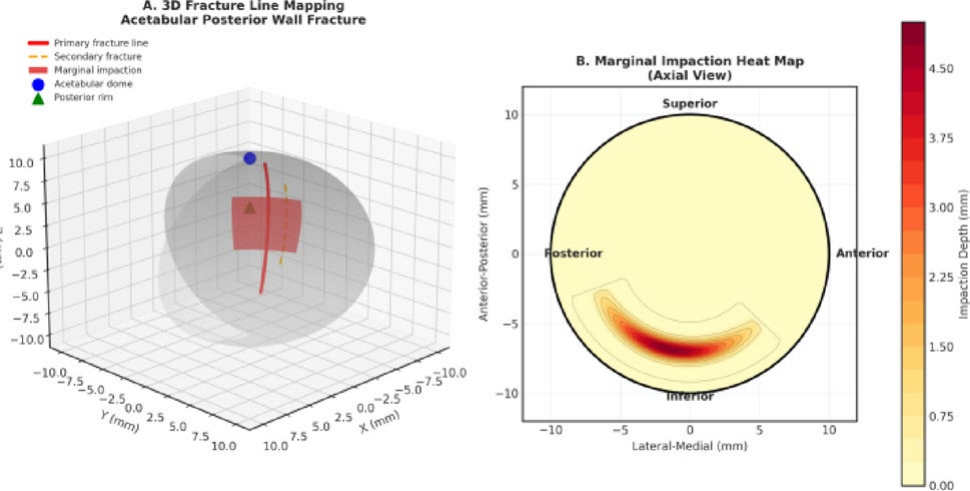
Patient demographics and injury characteristics

Twenty-two patients met the inclusion criteria during the study period. Patient demographics and injury characteristics are summarized in Table 1 and Fig. 3. The cohort comprised 15 males (68.2%) and 7 females (31.8%) with a mean age of 45.3 ± 16.8 years (range 23–74 years). Mean body mass index was 26.4 ± 3.8 kg/m^2^ (range 19.8–34.2 kg/m^2^). Motor vehicle accidents were the predominant mechanism of injury (68.2%), followed by falls from height (22.7%) and motorcycle accidents (9.1%). Associated hip dislocation was present in 9 patients (40.9%), with 2 patients (9.1%) sustaining concurrent sciatic nerve injury that recovered completely by final follow-up. Mean follow-up duration was 21.3 ± 6.4 months (range 12–30 months).

**Table 1:** Patient demographics and injury characteristics

Variable	Value
Age (years), mean ± SD	45.3 ± 16.8
Body mass index (kg/m^2^), mean ± SD	26.4 ± 3.8
Sex, n (%)	
Male	15 (68.2)
Female	7 (31.8)
Mechanism of injury, n (%)	
Motor vehicle accident	15 (68.2)
Fall from height	5 (22.7)
Motorcycle accident	2 (9.1)
Associated hip dislocation, n (%)	9 (40.9)
Sciatic nerve injury, n (%)	2 (9.1)

Three-dimensional fracture line mapping and marginal impaction distribution in posterior wall acetabular fractures. Panel A. Three-dimensional fracture line mapping of a posterior wall acetabular fracture demonstrating the spatial relationship between primary and secondary fracture lines, the marginal impaction zone, and key anatomical landmarks including the acetabular dome and posterior rim. Panel B. Axial-view heat map illustrating the distribution and depth of marginal impaction across the acetabular articular surface. Warmer colors indicate greater impaction depth, with predominant involvement of the posteroinferior region.

### Volumetric measurements and reliability

**Fig. 4:**
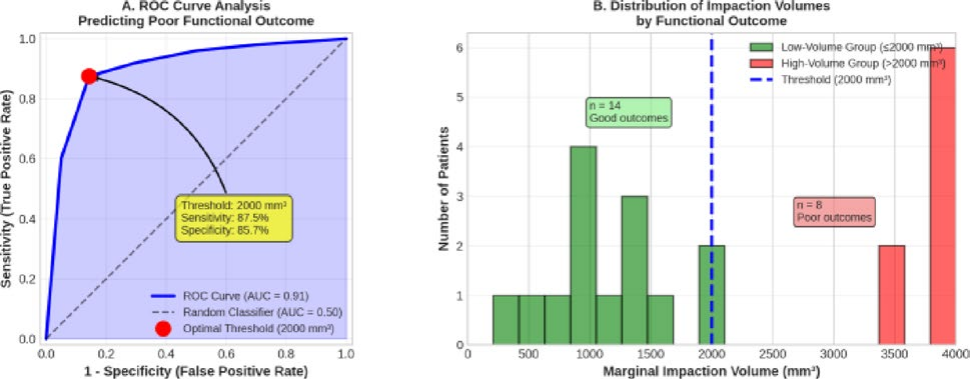
Marginal impaction volume distribution and ROC curve

The mean impacted fragment volume was 1,847.3 ± 1,124.6 mm^3^ (range 89–3,842 mm^3^, median 1,682 mm^3^). Mean acetabular volume was 36,248 ± 4,127 mm^3^ (range 28,940–43,865 mm^3^). The mean impaction volume-to-acetabular volume ratio was 5.12 ± 3.15% (range 0.24–10.43%). Distribution analysis revealed a right-skewed pattern with 36.4% of patients (n = 8) having volumes > 2000 mm^3^ (Fig. 4).

Reliability analysis demonstrated excellent agreement for impaction volume measurements: intraobserver ICC = 0.95 (95% CI: 0.87–0.99) and interobserver ICC = 0.92 (95% CI: 0.81–0.97). Bland–Altman analysis showed minimal systematic bias with 95% limits of agreement within ± 82 mm^3^. For acetabular volume measurements: intraobserver ICC = 0.96 (95% CI: 0.89–0.99) and interobserver ICC = 0.93 (95% CI: 0.82–0.98).

Receiver operating characteristic (ROC) analysis and distribution of marginal impaction volumes. Panel A Receiver operating characteristic (ROC) curve analysis evaluating the ability of marginal impaction volume to predict poor functional outcomes at 2-year follow-up. The analysis demonstrates excellent discriminatory performance, with an area under the curve (AUC) of 0.91. An optimal exploratory threshold of 2000 mm^3^ was identified, yielding a sensitivity of 87.5% and specificity of 85.7%. Panel B Distribution of marginal impaction volumes according to functional outcome categories. The dashed vertical line represents the identified threshold of 2000 mm^3^, illustrating the separation between patients with predominantly good outcomes below the threshold and those with predominantly poor outcomes above the threshold.

### Determination of volumetric threshold

ROC curve analysis was performed to identify an optimal exploratory threshold for impaction volume in predicting poor functional outcomes (defined as HHS < 80 or MMAS < 15 at 2 years). To our knowledge, this represents the first volumetric quantification of marginal impaction in acetabular fractures, as the existing literature exclusively employs linear measurements (step-off > 2 mm) without established volumetric thresholds. The ROC analysis identified 2000 mm^3^ as the exploratory threshold that maximized the Youden index (sensitivity + specificity—1). The area under the curve (AUC) was 0.91 (95% CI: 0.78–1.00, p < 0.001) with sensitivity 87.5% and specificity 85.7% at this threshold (Fig. 3).

### Functional outcomes

Patients were stratified into two groups based on the identified exploratory threshold: Low-volume group (≤ 2000 mm^3^): n = 14 patients; High-volume group (> 2000 mm^3^): n = 8 patients.

At 6-month follow-up, mean HHS was 79.8 ± 14.6 (range 48–95) and mean MMAS was 15.4 ± 2.9 (range 9–18). Significant differences were observed between groups (HHS: 87.2 ± 8.3 vs 66.4 ± 11.7, p < 0.001; MMAS: 17.3 ± 1.5 vs 12.1 ± 2.4, p < 0.001).

At 2-year follow-up, mean HHS was 81.5 ± 14.2 (range 52–97) and mean MMAS was 16.1 ± 2.8 (range 10–18). Overall functional outcomes were distributed as follows: excellent in 5 patients (22.7%), good in 7 patients (31.8%), fair in 5 patients (22.7%), and poor in 5 patients (22.7%) based on HHS criteria. Significant differences in functional outcomes were observed between groups (Table 2).

**Table 2:** Functional outcomes comparison between groups

Outcome measure	≤ 2000 mm^3^ (n = 14)	> 2000 mm^3^ (n = 8)	p-value
6-month outcomes			
Harris hip Score, mean ± SD	87.2 ± 8.3	66.4 ± 11.7	< 0.001
MMAS, mean ± SD	17.3 ± 1.5	12.1 ± 2.4	< 0.001
2-Year outcomes			
Harris hip Score, mean ± SD	88.5 ± 7.2	68.3 ± 10.8	< 0.001
MMAS, mean ± SD	17.6 ± 1.2	13.4 ± 2.3	< 0.001

### Correlation analysis

**Fig. 5:**
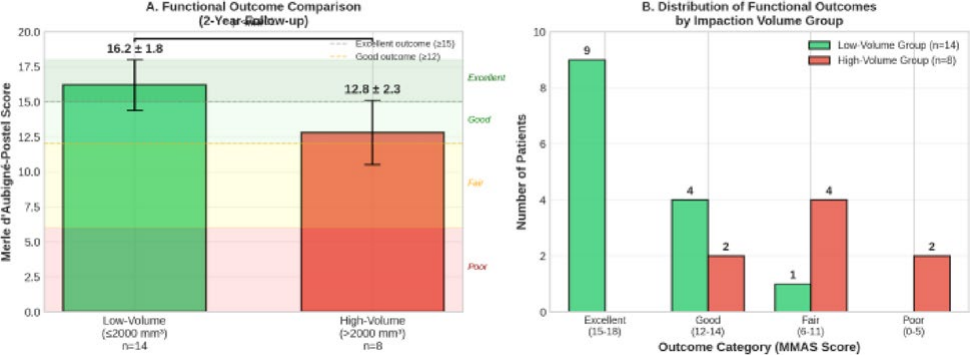
Correlation between impaction volume and functional outcomes

Strong negative correlations were identified between impaction volume and functional scores at 2 years (Fig. 5): HHS: r = -0.782 (p < 0.001); MMAS: r = -0.758 (p < 0.001). The impaction volume-to-acetabular volume ratio showed similar correlations: HHS: r = -0.763 (p < 0.001); MMAS: r = -0.741 (p < 0.001).

Linear regression analysis revealed that each 100 mm^3^ increase in impaction volume was associated with a 0.92-point decrease in HHS at 2 years (95% CI: -1.3 to -0.5, p < 0.001) and a 0.18-point decrease in MMAS (95% CI: -0.26 to -0.10, p < 0.001). Each 1% increase in impaction volume-to-acetabular volume ratio was associated with a 3.48-point decrease in HHS (95% CI: -4.92 to -2.04, p < 0.001).

Correlation analysis between impaction volume and functional outcomes. Panel A Comparison of mean Modified Merle d’Aubigné–Postel Score (MMAS) between the low-volume (≤ 2000 mm^3^) and high-volume (> 2000 mm^3^) impaction groups. Patients in the low-volume group demonstrated significantly higher functional scores compared to those in the high-volume group.

Panel B Distribution of functional outcome categories (excellent, good, fair, and poor) based on MMAS scores according to impaction volume group, demonstrating a higher proportion of fair and poor outcomes in the high-volume group.

### Radiographic outcomes and complications

Surgical reduction quality according to Matta's criteria was anatomical in 12 patients (54.5%), imperfect in 8 patients (36.4%), and poor in 2 patients (9.1%). Patients with impaction volumes > 2000 mm^3^ had significantly lower rates of anatomical reduction (25.0% vs 71.4%, p = 0.033).

Post-traumatic osteoarthritis developed in 9 patients (40.9%) during the follow-up period, with significantly higher incidence in the high-volume group (75.0% vs 21.4%, p = 0.012). Moderate to severe osteoarthritis (Brooker grade ≥ 2) occurred in 6 patients (75.0%) with volumes > 2000 mm^3^ compared to 1 patient (7.1%) with volumes ≤ 2000 mm^3^ (p = 0.001).

Avascular necrosis developed in 4 patients (18.2%), all in the high-volume group (p = 0.024). Heterotopic ossification occurred in 6 patients (27.3%) with higher rates in the high-volume group (50.0% vs 14.3%, p = 0.068). Two patients (9.1%) from the high-volume group required conversion to total hip arthroplasty at 18 and 24 months due to severe post-traumatic arthritis (Table 3).

**Table 3:** Complications and clinical outcomes

Complication	≤ 2000 mm^3^ (n = 14)	> 2000 mm^3^ (n = 8)	*p*-value
Post-traumatic osteoarthritis, n (%)	3 (21.4)	6 (75.0)	0.012
Avascular necrosis, n (%)	0 (0.0)	4 (50.0)	0.024
Heterotopic ossification, n (%)	2 (14.3)	4 (50.0)	0.068
Conversion to THA, n (%)	0 (0.0)	2 (25.0)	0.125
Superficial infection, n (%)	2 (14.3)	1 (12.5)	1.000

THA = Total hip arthroplasty

## Discussion

This study presents, to our knowledge, an exploratory quantitative volumetric analysis of marginal impaction in acetabular posterior wall fractures, introducing a data-driven threshold of 2000 mm^3^ that appears to stratify patients into different prognostic categories and may offer improved discriminatory value compared to traditional qualitative assessment methods (Table 4).

**Table 4:** ROC curve analysis statistics

Parameter	Impaction volume	Volume ratio
Optimal threshold	2000 mm^3^	5.5%
Area under curve (AUC)	0.91	0.89
95% Confidence Interval	0.78–1.00	0.75–1.00
*p*-value	< 0.001	< 0.001
Sensitivity	87.5%	87.5%
Specificity	85.7%	82.1%

ROC = receiver operating characteristic. Analysis performed to identify optimal threshold for predicting poor functional outcomes (HHS < 80 or MMAS < 15 at 2 years)

The identification of 2000 mm^3^ as an exploratory threshold emerged from ROC curve analysis demonstrating excellent discriminatory ability (AUC = 0.91, 95% CI: 0.78–1.00) with high sensitivity (87.5%) and specificity (85.7%). This threshold appeared to differentiate the groups in terms of outcomes: patients with impaction volumes ≤ 2000 mm^3^ demonstrated a mean Harris Hip Score of 87.2 at 2 years with only 7.1% experiencing fair or poor results, whereas those exceeding 2000 mm^3^ had significantly lower functional scores (HHS 66.4) with 87.5% experiencing fair or poor outcomes (p < 0.001). The threshold corresponds to approximately 5.5% of mean acetabular volume, which may allow future cross-population comparisons if validated. The volumetric nature captures three-dimensional complexity that cannot be adequately assessed through linear measurements alone, as traditional radiographic criteria fail to quantify total volume of comminuted bone requiring reconstruction [23, 24] Each 100 mm^3^ increase in impaction volume corresponds to a 0.92-point decrease in HHS, demonstrating a dose–response relationship supporting the clinical validity of volumetric quantification.

Marginal impaction has been recognized as a negative prognostic indicator since Letournel and Judet [25, 26], yet the literature has relied predominantly on qualitative descriptions. Keith et al.[27] and Calkins et al.[28] established that fragments involving more than 40–50% of posterior wall were unstable, but focused on fragment size rather than impaction volume. Moed et al.[29] identified marginal impaction as contributing to poor outcomes with only 55% good or excellent results, but categorized impaction qualitatively without objective criteria. Kreder et al.[30] reported 3.8-fold increased risk of post-traumatic arthritis with marginal impaction. Our study advances this work by providing the first quantitative threshold consistently measurable across observers (inter-observer ICC = 0.92). The superior discriminatory performance (AUC = 0.91) surpasses traditional predictive models, including Bhandari et al.'s [31] multivariable model (AUC = 0.74).

Our functional outcomes at 2-year follow-up (54.5% good/excellent, 45.5% fair/poor) must be interpreted contextually. Gänsslen et al. [26] reported good or excellent functional outcomes in 62.5% of patients with acetabular posterior wall fractures; however, this rate markedly decreased in cases with associated marginal impaction, which was observed in 52% of the cohort. Our cohort was selected to include only measurable marginal impaction, representing a higher-severity subgroup. Briffa et al. [6] reported 68% satisfactory outcomes at 10-year follow-up, identifying impaction as an independent predictor of failure. Tannast et al. [32] demonstrated 20-year survivorship free from total hip arthroplasty was only 43% in 810 patients. Our high-volume group outcomes suggest these patients are on a trajectory toward early arthroplasty conversion.

The clinical value of three-dimensional CT imaging in the assessment of acetabular fractures has been widely recognized [33, 34] and our volumetric analysis using 3D Slicer [16] extends beyond classification to provide quantitative prognostic information. The fracture mapping methodology pioneered by Armitage et al.[35] and Cole et al.[36] demonstrated that three-dimensional analysis reveals consistent injury patterns. Our volumetric quantification adds a functional dimension—quantifying bone loss that predicts clinical outcomes. Excellent reproducibility (intra-observer ICC = 0.95, inter-observer ICC = 0.92) suggests this methodology could be readily adopted by other centers.

The quantitative volumetric threshold may have potential clinical implications. Preoperative volumetric assessment could provide additional objective information for surgical planning and patient counseling, allowing clinicians to inform patients with impaction volumes exceeding 2000 mm^3^ that they may be at higher risk for less favorable outcomes. The threshold may guide decisions regarding advanced reconstructive techniques, particularly structural bone grafting [10, 37, 38]. Patients with large impaction volumes may benefit from modified rehabilitation protocols with extended protected weight-bearing [39] Volumetric data can guide surveillance strategies, with patients exceeding the threshold warranting intensified radiographic surveillance to identify candidates for early arthroplasty conversion [40].

Future research should aim to expand the proposed volumetric assessment approach by applying it to larger, multicenter, and prospectively collected patient cohorts to improve generalizability and external validity. Standardization of CT acquisition parameters and segmentation protocols will be essential to ensure reproducibility across different institutions and observers. In addition, extending this methodology to other acetabular fracture patterns may help determine its broader applicability and clinical relevance. With further validation, quantitative volumetric assessment of marginal impaction could be integrated into preoperative risk stratification models and surgical planning algorithms, potentially supporting more individualized treatment strategies.

Several important limitations of this study should be acknowledged. First, the retrospective and single-center design inherently restricts generalizability. In addition, although this cohort represents a well-defined and homogenous patient series, the modest sample size (n = 22) limits the statistical power of the analyses and reduces the robustness of subgroup comparisons. The relatively short follow-up period (mean 21.3 months) is also noteworthy, as post-traumatic osteoarthritis and avascular necrosis often evolve over longer periods; extended follow-up including long-term functional and activity-level data would provide a more comprehensive understanding of patient outcomes. Furthermore, focusing exclusively on posterior wall fractures restricts the applicability of the results to other acetabular fracture patterns.

Another limitation is the inability to incorporate patient-specific factors such as activity level, functional demand, and bone mineral density, all of which may influence clinical outcomes and complication risks. Additionally, although inter- and intra-observer reliability for volumetric measurements was excellent, the manual segmentation process may still introduce minor measurement variability. Finally, while 3D Slicer offers high-quality volumetric reconstructions, reproducibility across centers may be affected by differences in CT acquisition parameters and imaging protocols.

## Conclusion

This study demonstrates that quantitatively measured marginal impaction volume, obtained through three-dimensional CT analysis, is strongly associated with functional outcomes in patients with posterior wall acetabular fractures. Larger impaction volumes were correlated with significantly worse functional scores, higher rates of post-traumatic osteoarthritis, and an increased risk of adverse clinical outcomes. The exploratory threshold of 2000 mm^3^ identified in this cohort appears to stratify patients into distinct risk groups and may provide clinically meaningful information beyond traditional qualitative or linear assessment methods.

Given the limited sample size, this study should be considered a pilot investigation, and the proposed volumetric threshold requires validation in larger, multicenter prospective studies before routine clinical application. Nevertheless, quantitative volumetric assessment of marginal impaction may represent a promising tool for objective risk stratification, surgical planning, and patient counseling in posterior wall acetabular fractures.

## Data Availability

The data that supports the findings of this study are available on reasonable request from the corresponding author.
